# 

**Published:** 1881-09

**Authors:** 


### CONTENTS.

| | PAGE. |
|---|---|
| Malaria | 325 |
| A Ship's Log | 326 |
| The Good Old Times | 327 |
| In the West Indies | 328 |
| Niagara Falls | 329 |
| Is Alcohol Food ? | 330 |
| The Milky Way | 331 |
| Apoplexy | 333 |
| Blinders | 336 |
| Health and Disease | 337 |
| The Emotions | 352 |
| Snuff Taking | 352 |
| Why Persons Snore | 353 |
| Relations of Science to Speculation | 355 |
| Facts for the Curious | 357 |
| When to Feed Grain to Horses | 358 |
| Water from the Rock | 359 |
| The Mystery of Color Photography | 360 |
| Scientific Scraps | 360 |
| Sanitary Department of Hall's Journal | 361 |

